# A Medical Research Council randomized trial of single agent carboplatin versus etoposide and cisplatin for advanced metastatic seminoma

**DOI:** 10.1054/bjoc.2000.1498

**Published:** 2000-12

**Authors:** A Horwich, R T D Oliver, P M Wilkinson, G M Mead, S J Harland, M H Cullen, J T Roberts, S D Fossa, D P Dearnaley, E Lallemand, S P Stenning

**Affiliations:** Radiotherapy Unit, Royal Marsden Hospital, Downs Rd, Sutton, Surrey; School of Medicine, St Bartholomew's Hospital, West Smithfield, London; Christie Hospital, Wilmslow Rd, Withington, Manchester; Medical Oncology Dept, Royal South Hants Hospital, Brintons Terrace, Southampton; Department of Oncology, Middlesex Hospital, Mortimer St, London, W1N 8AA; Birmingham Oncology Centre, Queen Elizabeth Hospital, Edgbaston, Birmingham, B15 2TH; Northern Centre For Cancer Treatment, Newcastle General Hospital, Westgate Rd, Newcastle-upon-Tyne, NE4 6BE, UK; Norwegian Radium Hospital, Montebello 0310, Oslo 3, Norway; MRC Cancer Trials Office, 222 Euston Road, London, NW1 2DA, UK

**Keywords:** seminoma, germ cell tumour, chemotherapy, cisplatin, carboplatin, randomized control trial

## Abstract

The UK Medical Research Council conducted this trial of carboplatin chemotherapy in advanced seminoma to compare single agent carboplatin with a standard combination of etoposide with cisplatin. The use of single agent carboplatin was expected to be associated with reduced toxicity. A total of 130 patients with advanced seminoma were randomly assigned to treatment with either single agent carboplatin (C) at a dose of 400 mg/m^2^ to be corrected for glomerular filtration rate outside the range 81–120 ml min^–1^ and to be administered on day 1 of a 21 day cycle to a total of 4 cycles or to etoposide + platinum (EP). The trial was designed as an equivalence study aiming to exclude a reduction in the 3-year progression-free survival in patients allocated to carboplatin of between 10 and 15%, requiring initially a target accrual of 250 patients (90% power significance level 5% (one-sided)). The trial closed after 130 patients had been randomized following recommendation by an independent data monitoring committee. At a median follow-up time of 4.5 years, 81% of patients had been followed up for at least 3 years and 19 patients have died. The estimated PFS rate (95% Confidence Intervals (CI)) at 3 years was 71% (60–82%) in patients allocated C and 81% (71–90%) in those allocated EP; the 95% CI for the difference in 3 year PFS was – 6% to +19%. The hazard ratio of 0.64 (95% CI 0.32–1.28) favoured EP but the difference was not statistically significant (log rank chi-squared = 1.59 *P* = 0.21). The 3-year survival rate was 84% (75–92%) in those allocated C, and 89% (81–96%) in those allocated EP. The hazard ratio for survival was 0.85 with 95% CI, 0.35–2.10, log rank chi-squared = 0.12, *P* = 0.73. The trial has not demonstrated statistically significant differences in the major survival endpoints comparing single agent carboplatin with a combination of etoposide + cisplatin. This cannot be taken as an indication of equivalence since the limited size of this trial rendered it unable to exclude a 19% lower progression-free survival and survival in those treated with single agent carboplatin which would be important clinically. Standard initial chemotherapy for advanced seminoma should be based on cisplatin combinations and the role of carboplatin awaits the outcome of further studies. © 2000 Cancer Research Campaign http://www.bjcancer.com


*British Journal of Cancer* (2000) **83**(12), 1623–1629
© 2000 Cancer Research Campaign
doi: 10.1054/ bjoc.2000.1498, available online at http://www.idealibrary.com on IDEAL®

### A Medical Research Council randomized trial of single agent carboplatin versus etoposide and cisplatin for advanced metastatic seminoma

**A Horwich[1], RTD Oliver[2], PM Wilkinson[3], GM Mead[4], SJ Harland[5], MH Cullen[6], JT Roberts[7], SD Fossa[8], DP Dearnaley[1], E Lallemand[9] and SP Stenning[9] for the MRC Testicular Tumour Working Party**

[1]Radiotherapy Unit, Royal Marsden Hospital, Downs Rd, Sutton, Surrey; [2]School of Medicine, St Bartholomew's Hospital, West Smithfield, London; [3]Christie Hospital, Wilmslow Rd, Withington, Manchester; [4]Medical Oncology Dept, Royal South Hants Hospital, Brintons Terrace, Southampton; [5]Department of Oncology, Middlesex Hospital, Mortimer St, London W1N 8AA; [6]Birmingham Oncology Centre, Queen Elizabeth Hospital, Edgbaston, Birmingham B15 2TH; [7]Northern Centre For Cancer Treatment, Newcastle General Hospital, Westgate Rd, Newcastle-upon-Tyne, NE4 6BE, UK; [8]Norwegian Radium Hospital, Montebello 0310, Oslo 3, Norway; [9]MRC Cancer Trials Office, 222 Euston Road, London NW1 2DA, UK

**Summary** The UK Medical Research Council conducted this trial of carboplatin chemotherapy in advanced seminoma to compare single agent carboplatin with a standard combination of etoposide with cisplatin. The use of single agent carboplatin was expected to be associated with reduced toxicity. A total of 130 patients with advanced seminoma were randomly assigned to treatment with either single agent carboplatin (C) at a dose of 400 mg/m$^2$ to be corrected for glomerular filtration rate outside the range 81–120 ml min$^{-1}$ and to be administered on day 1 of a 21 day cycle to a total of 4 cycles or to etoposide + platinum (EP). The trial was designed as an equivalence study aiming to exclude a reduction in the 3-year progression-free survival in patients allocated to carboplatin of between 10 and 15%, requiring initially a target accrual of 250 patients (90% power significance level 5% (one-sided)). The trial closed after 130 patients had been randomized following recommendation by an independent data monitoring committee. At a median follow-up time of 4.5 years, 81% of patients had been followed up for at least 3 years and 19 patients have died. The estimated PFS rate (95% Confidence Intervals (CI)) at 3 years was 71% (60–82%) in patients allocated C and 81% (71–90%) in those allocated EP; the 95% CI for the difference in 3 year PFS was – 6% to +19%. The hazard ratio of 0.64 (95% CI 0.32–1.28) favoured EP but the difference was not statistically significant (log rank chi-squared = 1.59 $P$ = 0.21). The 3-year survival rate was 84% (75–92%) in those allocated C, and 89% (81–96%) in those allocated EP. The hazard ratio for survival was 0.85 with 95% CI, 0.35–2.10, log rank chi-squared = 0.12, $P$ = 0.73. The trial has not demonstrated statistically significant differences in the major survival endpoints comparing single agent carboplatin with a combination of etoposide + cisplatin. This cannot be taken as an indication of equivalence since the limited size of this trial rendered it unable to exclude a 19% lower progression-free survival and survival in those treated with single agent carboplatin which would be important clinically. Standard initial chemotherapy for advanced seminoma should be based on cisplatin combinations and the role of carboplatin awaits the outcome of further studies. © 2000 Cancer Research Campaign http://www.bjcancer.com

**Keywords:** seminoma; germ cell tumour; chemotherapy; cisplatin; carboplatin; randomized control trial

The development of chemotherapy for seminoma has tended to follow the principles determined for the management of non-seminoma, a more common clinical indication (Einhorn and Williams, 1980). Variations of a combination of cisplatin, etoposide and bleomycin (BEP) have become standard treatment, not only for patients with disseminated seminoma or recurrence after radiotherapy, but also for those who present with metastatic disease confined to the retroperitoneal nodes but which measures more than 5 cm in transverse diameter (Horwich and Dearnaley, 1992; Mencel et al, 1994; Fosså and Horwich, 1997). The role of bleomycin in the treatment of seminoma with these drug combinations is uncertain, firstly, in view of the poor results of treatment with the combination of vinblastine and bleomycin (Samuels et al, 1976) and secondly, in view of the demonstrated efficacy of combinations such as etoposide plus cisplatin (Mencel et al, 1994) or cyclophosphamide plus cisplatin (Logothetis et al, 1987). Furthermore, a number of reports emphasize the sensitivity of seminoma to single agent cisplatin or carboplatin suggesting that these approaches lead to similar disease control to that achieved by combination chemotherapy but with reduced toxicity (Samuels and Logothetis, 1983; Horwich et al, 1989; Oliver, 1988; Dieckmann et al, 1990; Schmoll et al, 1991). The UK Medical Research Council therefore launched a prospective randomized trial in 1990 to compare single agent carboplatin (C) with the combination of etoposide and cisplatin (EP) in patients with advanced metastatic seminoma.

*Received 31 March 2000*
*Revised 26 July 2000*
*Accepted 15 August 2000*

*Correspondence to:* A Horwich

### PATIENTS AND METHODS

### Eligibility and study entry

Chemo-naive patients with histologically confirmed testicular or extragonadal seminoma, glomerular filtration rate ≥40 ml min$^{-1}$, and AFP <25 ku l$^{-1}$, who had either relapsed with any stage of disease following previous irradiation, or were newly diagnosed with Royal

Marsden stage IIC, III or IV disease were eligible (Peckham et al, 1979). Patients were randomized by telephone call to the MRC Cancer Trials Office, and allocated either 4 cycles of etoposide and cisplatin (EP) or 4 cycles of single agent carboplatin (C). Treatment was allocated by minimization balanced by centre and stage.

### EP schedule and dose modifications

For patients allocated EP, the recommended schedule was pre-hydration of 1 l N-saline plus 20 mmol KCl six-hourly × 2; after which cisplatin at 20 mg/m$^2$ was given in 1 l N-saline + 20 mmol KCl over 6 hours, followed by 1 l N-saline + 20 mmol KCl 6 hourly × 3. Cisplatin was given at this dose on days 1, 2, 3, 4 and 5 of each cycle. Etoposide was given at 120 mg/m$^2$ as a 1 hour infusion in 250 ml N-saline on days 1, 2 and 3. Four cycles of EP were given at 21-day intervals.

Dose modifications for etoposide were based on full blood counts on day 1 of the cycle to be administered. Full doses were given only if WBC was $>2 \times 10^9$ l$^{-1}$ and platelets $>90 \times 10^9$ l$^{-1}$. If WBC was $<1 \times 10^9$ l$^{-1}$ or platelets were $<50 \times 10^9$ l$^{-1}$, chemotherapy was delayed for 4 days and recommenced with 50% etoposide when WBC and platelets had risen. For blood counts between these levels, or for nadir WBC $<1.5 \times 10^9$ l$^{-1}$ or nadir platelets $<50 \times 10^9$ l$^{-1}$, etoposide dose was reduced by 25%. If glomerular filtration rate (GFR) measured by clearance of creatinine or EDTA fell below 40 ml min$^{-1}$, cisplatin was discontinued and treatment with carboplatin recommended.

### Carboplatin schedule and dose modifications

For patients allocated carboplatin, a single dose was given over 60 minutes in 500 ml 5% dextrose on day 1 of each cycle. Four cycles were given at 21-day intervals. The initial dose of carboplatin was based on Glomerular Filtration Rate (GFR) estimated by clearance of Chromium-51 labelled EDTA, and surface area, as follows (If GFR was estimated by creatinine clearance the carboplatin dose in this table was reduced by 10%.)

| EDTA GFR | Carboplatin dose (mg/m$^2$) |
|---|---|
| >140 | 460 |
| 121–140 | 440 |
| 81–120 | 400 |
| 61–80 | 350 |
| 41–60 | 300 |
| 26–40 | 200 |
| <25 | 100 |

Blood counts were measured weekly during chemotherapy. Dose modifications for subsequent carboplatin were based on nadir blood counts; the dose was escalated by 10% if nadir WBC was $>3 \times 10^9$ l$^{-1}$ and platelets $>150 \times 10^9$ l$^{-1}$, and reduced by 10% if WBC was $<1.5 \times 10^9$ l$^{-1}$ or platelets $<50 \times 10^9$ l$^{-1}$. However if, on day 1 of the cycle to be administered, WBC was $<2 \times 10^9$ l$^{-1}$, or platelets $<100 \times 10^9$ l$^{-1}$ the course was delayed and blood counts reassessed every 3 days until they exceeded these levels.

### Treatment of residual masses

The protocol recommended that residual masses greater than 5 cm were biopsied and if tumour-negative, followed up with CT scans at 6 and 12 months, and annually thereafter, unless complete regression occurred. Masses less than 5 cm in diameter were followed by CT scanning at 3 monthly intervals for the 1st year, and considered for biopsy if no regression had occurred after 6 months. Patients with a tumour-negative biopsy had a further CT at 12 months and then annually. A tumour-positive biopsy at any time was recorded as a treatment failure, with subsequent treatment left to clinical discretion. In practice only 3 of the 10 patients who had residual masses over 5 cm in size were biopsied; the remainder were followed as for smaller masses.

Following recurrence, patients were re-staged with serum marker assays and CT of thorax and abdomen. Treatment was left to clinical discretion, but with cisplatin-based regimens being recommended for carboplatin relapses, and consideration given to the possibility of radiotherapy consolidation.

### Statistical considerations

The primary endpoint of the study was progression-free survival (PFS) at 3 years. The enlargement of existing masses, appearance of new metastases, tumour-positive biopsy at any time and death from tumour or treatment were considered as events in the PFS analysis. Three-year PFS after EP was expected to be approximately 80%, and the trial was designed as an equivalence study, aiming to exclude a reduction in the 3-year PFS in patients allocated carboplatin of between 10 and 15%. The initial study target was 250 patients, sufficient to exclude a 15% difference (90% power, significance level (1-sided) 5%), with continuation to 550 (sufficient to exclude a 10% difference) dependent upon the Data Monitoring Committee (DMC) recommendation at that time. Secondary endpoints included failure-free survival in which death from any cause, as well as those events described for PFS were included, overall survival, acute toxicity and an optional assessment of long-term ototoxicity and fertility. All event-free rates and confidence intervals on 3-year survival rates were calculated using the Kaplan–Meier method, and compared using the log-rank test. The event hazard ratios and their 95% confidence intervals were estimated using Cox's proportional hazards regression model; hazard ratios less than one indicate a benefit to the etoposide/cisplatin combination.

### RESULTS

### Accrual and patient characteristics

The study opened in August 1990, and by November 1993, 125 patients had been randomized. At this time, the independent DMC met to review the interim results. Accrual had declined considerably in the previous 6 months following presentation of trial results confirming the inferiority of carboplatin-based therapy over cisplatin-based chemotherapy for metastatic non-seminoma. The DMC recommended that the trial be closed in view of the poor recent accrual and the interim results which, though not conventionally significant, showed similar trends to the results of randomized trials in metastatic non-seminoma (Bajorin et al, 1993; Horwich et al, 1997) and non-randomized studies in metastatic seminoma (C-13 events, EP 7 events, Hazard Ratio 0.45 log-rank, $P = 0.08$). Events were failures or early death. The trial was closed formally early in 1994, when 130 patients had been randomized from 18 centres in the UK, 5 in the Netherlands and 1 in Norway. 64 patients were allocated C, and 66 EP. We report here long-term follow-up of these patients.

 *© 2000 Cancer Research Campaign*

**Table 1** Pre-treatment patient characteristics

| | | Treatment Allocated | | | |
|---|---|---|---|---|---|
| | | Carboplatin | | EP | |
| | | No. | % | No. | % |
| Relapse after | No | 55 | 85.9 | 57 | 86.4 |
| radiotherapy | Yes | 9 | 14.1 | 9 | 13.6 |
| Site of primary | Testis | 53 | 84.1 | 57 | 86.4 |
| | Mediastinium | 3 | 4.8 | 3 | 4.5 |
| | Abdomen | 4 | 6.3 | 5 | 7.5 |
| | Other | 3 | 4.8 | 1 | 1.5 |
| HCG (IU/1) at entry | <10 | 49 | 76.6 | 39 | 60.9 |
| | 11–200 | 15 | 23.4 | 25 | 39.1 |
| | Not known | – | – | 2 | – |
| Abdominal nodes | None | 15 | 23.8 | 11 | 17.5 |
| (max diameter in cm) | <2 | 1 | 1.5 | 1 | 1.6 |
| | 2–5 | 6 | 9.5 | 8 | 12.7 |
| | 5–10 | 28 | 44.4 | 33 | 52.4 |
| | >10 | 13 | 20.6 | 10 | 15.9 |
| | Not known | 1 | – | 1 | – |
| Mediastinal nodes | None | 46 | 76.7 | 50 | 78.1 |
| (max diameter in cm) | <2 | | | 3 | 4.7 |
| | 2–5 | 8 | 13.3 | 4 | 6.3 |
| | 5–10 | 3 | 5.0 | 4 | 6.3 |
| | >10 | 3 | 5.0 | 3 | 4.7 |
| | Not known | 4 | – | 2 | – |
| Neck nodes | None | 53 | 82.8 | 61 | 93.8 |
| (max diameter in cm) | <2 | 1 | 1.6 | | |
| | 2–5 | 8 | 12.5 | 4 | 6.2 |
| | 5–10 | 2 | 3.1 | | |
| | Not known | – | – | 1 | – |
| Lung metastases | None | 59 | 93.7 | 61 | 93.8 |
| (number) | 1–4 | 4 | 6.3 | 3 | 4.6 |
| | >=10 | | | 1 | 1.5 |
| | Not known | 1 | | 1 | – |
| (max diameter in cm) | None | 59 | 93.7 | 61 | 95.3 |
| | 0.1–1.0 | 2 | 3.2 | 1 | 1.6 |
| | 1.1–2.0 | 1 | 1.6 | 1 | 1.6 |
| | 2.1–3.0 | 1 | 1.6 | | |
| | >3.0 | | | 1 | 1.6 |
| | Not known | 1 | – | 2 | – |
| Liver metastases | NO | 64 | 100.0 | 66 | 100.0 |
| Bone metastases | NO | 62 | 98.4 | 64 | 97.0 |
| | YES | 1 | 1.6 | 2 | 3.0 |
| | Not known | 1 | – | – | – |
| Brain metastases | NO | 63 | 98.4 | 66 | 100.0 |
| | YES | 1 | 1.6 | | |
| Total | | 64 | 100.0 | 66 | 100.0 |

Patient characteristics are summarized in Table 1, and were well balanced between the treatment groups. 18 patients (14%) had relapsed following previous radiotherapy. Of the remainder, 75 had stage IIC disease, 29 stage III and 8 stage IV. The primary tumour site was the testis in 85% of patients. Age at study entry ranged from 18 to 70 years with a median of 38.

### Treatment details

Of 64 patients allocated C, treatment information is missing on 4 patients. Of the remaining 60, 56 received all 4 cycles and in 26 of these patients at least one dose escalation was possible. 3 patients changed treatment before 4 cycles had been completed because of lack of response, including one patient in whom the diagnosis was later changed to malignant thymoma. Finally, one patient received BEP throughout (clinical decision).

Of 66 patients allocated EP, treatment information is missing on 2. Of the remaining 64, 62 received all 4 EP cycles, with 10 of these 62 patients requiring at least one dose reduction of etoposide because of haematological toxicity, and one requiring a 50% reduction in cycles 3 and 4 cisplatin because of renal and ototoxicity. One patient received carboplatin rather than cisplatin in cycles 3 and 4 because of renal toxicity, and one patient died after 2 cycles from a pulmonary embolism which was possibly treatment related.

The maximum toxicity grade over all chemotherapy cycles is given in Table 2. WBC was significantly lower in patients allocated EP (chi-square (trend) $P < 0.001$) with 20 (32%) EP patients having grade 3 neutropenia compared with 2 (3%) of those allocated C. Platelets were statistically significantly lower in C patients (chi-square (trend) $P = 0.02$), but grade 3 or 4 toxicity occurred in only 5 patients in each group. Three patients allocated

© 2000 Cancer Research Campaign

**Table 2** Maximum haematological toxicity

| | | Treatment allocated | | | |
|---|---|---|---|---|---|
| | | Carboplatin | | EP | |
| | | *N* | % | *N* | % |
| Thrombocytopenia | Grade 0 | 33 | 56.9 | 53 | 85.5 |
| | Grade 1 | 11 | 19.0 | 2 | 3.2 |
| | Grade 2 | 9 | 15.5 | 2 | 3.2 |
| | Grade 3 | 4 | 6.9 | 5 | 8.1 |
| | Grade 4 | 1 | 1.7 | | |
| Neutropenia | Grade 0 | 16 | 27.6 | 3 | 4.8 |
| | Grade 1 | 17 | 29.3 | 15 | 24.2 |
| | Grade 2 | 23 | 39.7 | 24 | 38.7 |
| | Grade 3 | 2 | 3.4 | 20 | 32.3 |
| Total | | 58 | 100.0 | 62 | 100.0 |

*N* = Number of patients.

**Table 3** Chemotherapy response evaluation

| | | Treatment Allocated | | | |
|---|---|---|---|---|---|
| | | Carboplatin | | EP | |
| | | *N* | % | *N* | % |
| Chemotherapy response | Complete response | 21 | 32.8 | 20 | 30.3 |
| | Partial response | 38 | 59.4 | 43 | 65.2 |
| | No response | 1 | 1.6 | 1 | 1.5 |
| | Progressive disease | 3 | 4.7 | 1 | 1.5 |
| | Not evaluable | 1 | 1.6 | 1 | 1.5 |

*N* = Number of patients.

C and 10 allocated EP had grade ≥ 1 WHO grade diarrhoea, while grade 3 or 4 nausea and vomiting was seen in 6 C patients and 10 EP patients. Neurotoxicity was assessed immediately after completion of chemotherapy in 29 C patients, none of whom experienced any WHO grade 1 or greater toxicity, and 38 EP patients 4 of whom had grade 1 and one grade 2. None of the 27 C patients assessed at the completion of chemotherapy had WHO grade 1 or more ototoxicity, but 6 EP patients did, with 4 going on to have high tone hearing loss (8 kHz) confirmed by audiometry. As expected, the number of in-patient days during initial chemotherapy was fewer in patients allocated C, with a median across all cycles of 3 days (range 0–12), while for EP the median was 20 days (range 10–30).

### Response

Of patients allocated C, 46 patients had a total of 59 residual masses (median diameter 2.5 cm, range 0.5 to 13.8 cm), the majority (39 patients) in the abdominal nodes. Two of these abdominal masses were biopsied immediately after chemotherapy (tumour-free) and 2 others were irradiated. Of patients allocated EP, 50 patients had a total of 57 residual masses the majority (42) again being the abdominal nodes (median diameter 2.5 cm, range 0.5 to 6 cm). One patient had resection of inguinal masses which were tumour-free, and 1 had biopsy of an abdominal mass revealing seminoma. 3 were residual masses in the testis; orchidectomy revealed no tumour. 3 of the 42 abdominal masses were irradiated soon after chemotherapy. The final response assessment (after any surgery or radiotherapy) is given in Table 3, in which patients with residual nodal masses less than 2 cm in diameter are reported as having complete response (according to the definitions used at the time).

### Follow-up and events

Median follow-up time is now $4\frac{1}{2}$ years. 19 patients have died, and of the survivors, 98 (81%) have been followed-up for at least 3 years. A total of 22 events (failures) have occurred among patients allocated carboplatin; 12 patients progressed but are currently alive and free from active disease, 9 after salvage chemotherapy and 3 after radiotherapy. Seven have died from germ cell tumour (GCT) following progression despite salvage chemotherapy, one has died with the cause unknown (presumed to be GCT) and 2 have died from other causes without progression (one from misdiagnosed malignant thymoma, one from bronchopneumonia and multiple infarct dementia). Thus a total of 19 patients have had progression or death from GCT and are included in the progression-free survival analysis, and 10 have died and are included in the overall survival analysis.

A total of 14 events (failures) have occurred among patients allocated EP. 5 patients are currently alive following successful treatment of progression (3 after chemotherapy, 2 after radiotherapy); 8 have died from GCT following progression despite salvage chemotherapy, and one died while on treatment from a pulmonary embolism and heart failure which was possibly treatment related. All 14 events have been included in the progression-free survival analysis, and the 9 deaths are included in the all-cause survival analysis.

The progression-free survival (PFS) curves are shown in Figure 1. The hazard ratio of 0.64, 95% CI (0.32, 1.28) favours the EP

 *© 2000 Cancer Research Campaign*

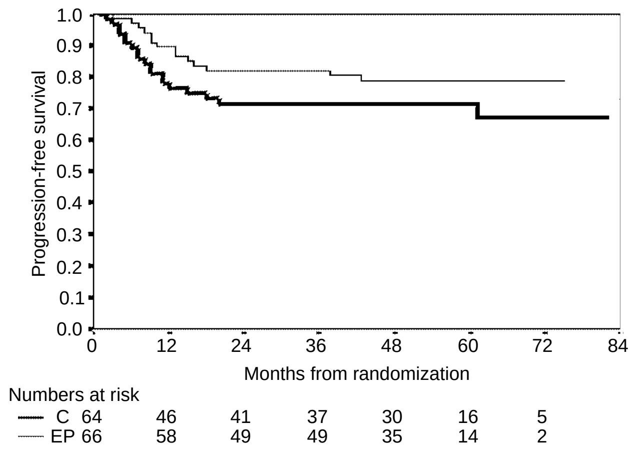

**Figure 1** Progression-free survival by allocated treatment. 3 year % PFS (95% CI) are for C, 71 (60–82); for EP 81 (71–90). Log rank $P$ = 0.21

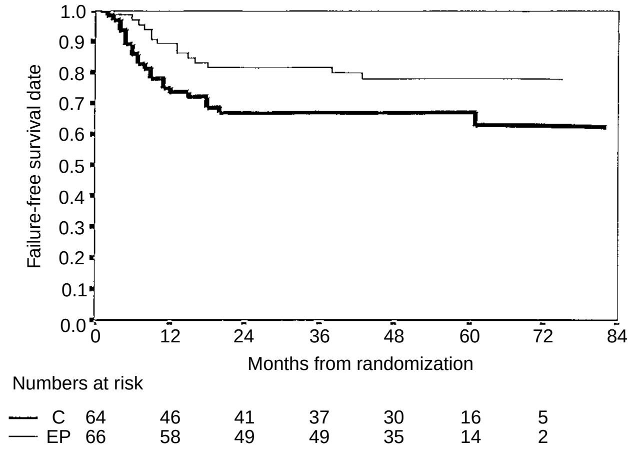

**Figure 2** Overall survival by allocated treatment. 3 year % S (95% CI) are for C 84 (75–92); for EP 89 (81–96). Log rank $P$ = 0.73

group, but the difference is not statistically significant (log-rank $\chi^2$ = 1.59, $P$ = 0.21). The estimated PFS rate at 3 years is 71%, 95% CI (60%, 82%) in patients allocated C and 81%, 95% CI (71%, 90%) in those allocated EP, the 95% CI for the difference in 3-year PFS being (– 6%, +19%).

For failure-free survival (FFS) the hazard ratio again favours the EP arm being 0.56, 95% CI (0.28, 1.09), log-rank $\chi^2$ = 3.02, $P$ = 0.08. The estimated FFS at 3 years is 67%, 95% CI (55%, 78%) in those allocated C and 81%, 95% CI (71%, 90%) in those allocated EP. Thus the difference in 3-year FFS is 14% with 95% CI (–2%, +22%).

Overall survival curves are given in Figure 2. The hazard ratio is 0.85, with a broad 95% CI (0.35, 2.10), log-rank $\chi^2$ = 0.12, $P$ = 0.73. The 3-year survival rate is 84% (75%, 92%) in those allocated C and 89% (81%, 96%) in those allocated EP. The estimated difference in 3-year survival is therefore 5% in favour of EP, but with a 95% CI (–20%, +10%).

### DISCUSSION

A pilot study of single agent carboplatin chemotherapy for advanced seminoma was based on 70 patients treated between 1982 and 1990 at the Royal Marsden (Horwich et al, 1989). This documented the low toxicity of this approach and no patients suffered neurotoxicity, ototoxicity or significant renal damage. There was only one episode of neutropenic sepsis and no thrombocytopenic bleeding. With a median follow up of 3 years, the actuarial 3-year relapse-free survival was 77% and the cause-specific survival was 94%. Of the 16 patients who relapsed, 12 were successfully salvaged with combination chemotherapy leading to an overall level of survival equivalent to that obtained with initial cisplatin-based combination chemotherapy. These results were supported by early results of phase II studies of single agent carboplatin from Germany (Dieckmann et al, 1990; Schmoll et al, 1991) and formed the basis of the decision to launch a trial to compare progression-free survival after either carboplatin or the combination of etoposide, cisplatin. It was anticipated that 250 patients should be recruited in order to exclude a 15% reduction in progression-free survival. However, following recruitment of 125 patients, the trial was closed on the advice of an independent data monitoring committee on the basis of results of carboplatin in non-seminoma, slowing recruitment and a trend towards inferior results on the carboplatin arm. The trial was thus under-powered to determine the possibility of significant differences in major survival endpoints between the two arms and though the progression-free survival at 3 years was 10% lower after single agent carboplatin, the 95% confidence intervals on the difference is –6% to +19%. As a consequence of salvage treatment, the difference in overall survival was even less at 5% in favour of EP but with 95% confidence intervals ranging from –20% to +10%.

No other randomized trials focusing solely on seminoma have yet been reported fully, although a German trial of single agent carboplatin versus etoposide ifosfamide cisplatin has been reported in abstract (Clemm et al, 2000), to show inferior disease control by single agent carboplatin, but no difference in overall survival. Seminoma patients have often been included with good prognosis non-seminoma for the purposes of randomized trials (Einhorn et al, 1989; Bajorin et al, 1993; Loehrer et al, 1995) but rarely in sufficient numbers to permit meaningful treatment comparisons. The most useful randomized data comes from the randomized trial of etoposide/cisplatin (EP) versus etoposide/carboplatin (EC) in good prognosis metastatic germ cell tumours (Bajorin et al, 1993). This trial included 64 patients with histologically pure seminoma and normal AFP. 27 of 31 patients randomized to EP achieved a complete response, with 2 of the 4 who failed to do so suffering early death. No relapses occurred with a median follow-up of 22 months, and so a durable response rate of 87% was reported. Of 33 patients allocated EC, 2 failed to achieve a complete response and 4 patients (13%) relapsed following a complete response. The durable response rate was therefore 82%. These regimens included Etoposide at 100 mg per $m^2$ per day for 5 days, a higher dose than in our trial; the outcomes were similar but small numbers and the risk of selection has precluded a conclusion on optimal etoposide dose.

A retrospective analysis was performed on 143 patients with advanced seminoma treated at the Memorial Sloan Kettering Cancer Center (MSKCC) (Mencel et al, 1994). In a non-randomized comparison, durable response rates of 79% were reported for the 43 patients treated with the VAB-6 regimen, 92% in 60 patients treated with EP, and 83% in 35 patients treated with EC. Again in a non-randomized comparison, 3-year progression-free survival rates were reported by the MRC (Fosså et al, 1997) in a publication reporting prognostic factors in metastatic seminoma. The database

*© 2000 Cancer Research Campaign*

included 58 patients treated with C, with a 3-year PFS of 79%, 15 treated with single agent cisplatin with 100% 3-year PFS, and 69 patients treated with BEP, in whom the 3-year PFS was 88%. As with the MSKCC study, patient characteristics were not reported separately for each treatment group, and so the confounding influence of an imbalance in such factors cannot readily be assessed.

Our protocol did not specify a requirement for treatment of any residual masses immediately following chemotherapy since these are particularly common after treatment of bulky seminomatous masses and usually do not contain persisting malignancy (Horwich and Dearnaley, 1992). There is little evidence for routine adjuvant treatment after combination chemotherapy (Schultz et al, 1989; Duchesne et al, 1997). It has been suggested that residual masses more than 3 cm in diameter early in the period following chemotherapy may be at greater risk of containing viable seminoma (Motzer et al, 1988; Puc et al, 1996) and the protocol therefore recommended a biopsy of any residual mass more than 5 cm in diameter. In this trial, only 5 residual masses in 4 patients in this time period were resected (1) or biopsied (3) and one revealed viable seminoma. 96 patients had residual masses of whom 89 were managed by observation; a detailed failure analysis of these patients is the subject of a separate analysis and report.

The efficacy of carboplatin noted in phase II studies of the treatment of advanced seminoma has led to its evaluation in the role of adjuvant therapy post-orchidectomy in stage I seminoma as an alternative to retroperitoneal lymph node irradiation (Oliver et al, 1994). Oliver and colleagues found that of 54 patients with stage I seminoma treated with 2 cycles of adjuvant carboplatin, there were 2 recurrences after a median follow up of 62 months and of 65 patients with stage I seminoma, treated with a single cycle of adjuvant carboplatin, there were no recurrences after a median follow up of 20 months. This has led to a current trial coordinated through the UK Medical Research Council (TE19) to evaluate carboplatin in the adjuvant treatment of stage I seminoma by prospective randomized comparison with adjuvant radiotherapy.

Current levels of evidence do not provide a sufficiently secure basis to recommend single agent carboplatin for the chemotherapy of advanced metastatic seminoma. The PFS we found after carboplatin of 71% at 3 years, was not significantly different from the 81% found after EP, and more patients relapsing after carboplatin were salvaged (12/19 compared to 5/14) leading to very similar levels of mortality (7 or 8 after carboplatin; 8 after EP) from progressive GCT. The lack of significant differences in outcomes in this trial comparing single agent carboplatin with etoposide and cisplatin may be a consequence of the low power of the trial to determine clinically important differences between the treatments. The ongoing trial from the German Testicular Cancer Group, comparing single agent carboplatin with the combination of etoposide, ifosphamide and cisplatin will contribute to this judgement. A recent prognostic factor analysis based on 236 patients with advanced seminoma treated with cisplatin-based chemotherapy at 10 European Oncology Units (Fosså et al, 1997) identified a good prognosis group comprising patients who had not been treated previously with radiotherapy, and who had either stage II seminoma with any serum lactate dehydrogenase (LDH) level at presentation or stage III and IV patients without non-pulmonary visceral metastases whose serum LDH was less than 2 × the upper limit of normal. These patients had a 94% 3-year progression-free survival (PFS). The poor prognostic group included all other patients and had a 56% 3-year progression-free survival (Fosså et al, 1997).

Our results are consistent with this classification. In the 94 patients who could be classified, 86 would be in the good prognosis group and had a 3-year PFS probability of 84% (95% CI, 80–88%) and 8 would be in the poor prognosis group with a 3-year PFS probability of 44% (95% CI, 8–80%).

The International Germ Cell Cancer Collaborative Group (IGCCCG) classification of prognosis in metastatic seminoma (International Germ Cell Cancer Collaborative Group, 1997) identified a good prognosis group without non-pulmonary visceral metastases with a 5-year PFS rate of 82% and an ‘intermediate prognosis’ group, who did have non-pulmonary visceral metastases, with a 5-year PFS rate of 67%. On this classification patients in our trial with good prognosis ($n$ = 125) had a 77% PFS at 3 years (95% CI, 69–84%) and those 5 with intermediate prognosis had a 3-year PFS rate of 60% (95% CI, 20–99%).

A reasonable recommendation for standard practice would be to treat a good prognosis group with the combination of etoposide and cisplatin, and to regard carboplatin as a reasonable alternative for those few patients unable to tolerate cisplatin due to co-morbid disease. For the small number of patients who present with poor risk factors more intensive treatment using bleomycin or an alternative agent in combination with EP or entry into appropriate phase III studies which include NSGCT patients would be appropriate.

### ACKNOWLEDGEMENTS

This work was undertaken by a number of NHS Trusts who received a proportion of their funding from the NHS Executive; the views expressed in this publication are those of the authors and not necessarily those of the NHS Executive. This work was also supported by the Institute of Cancer Research, and the Medical Research Council.

### REFERENCES

Bajorin DF, Sarosdy MF, Pfister DG et al (1993) Randomised trial of etoposide and cisplatin versus etoposide and carboplatin in patients with good-risk germ cell tumours: A Multi-institutional Study. *J Clin Oncol* **11**: 598–606

Clemm C, Bokemeyer C, Gerl A et al (2000) Randomized trial comparing cisplatin/etoposide/ifosfamide with carboplatin monochemotherapy in patients with advanced metastatic seminoma. *Proc ASCO* **19**: 326a

Dieckmann K-P, Bornhoeft G and Huland H (1990) Ambulante carboplatin-monotherapie beim fortgeschrittenen seminom. *Urologe A* **29**: 281–285

Duchesne GM, Stenning SP, Aass N et al (1997) Radiotherapy after chemotherapy for metastatic seminoma – a diminishing role. MRC Testicular Tumour Working Party. *Eur J Cancer* **33**: 829–835

Einhorn LH and Williams SD (1980) Chemotherapy of disseminated seminoma. *Cancer Clin Trials* **3**: 307–313

Einhorn LH, Williams SD, Loehrer PJ (1989). Evaluation of optimal duration of chemotherapy in favourable-prognosis disseminated germ cell tumors: a Southeastern Cancer Study Group protocol. *J Clin Oncol* **7**: 387–391

Fosså SD and Horwich A (1997) Current status of chemotherapy in advanced seminoma [editorial]. *Eur J Cancer* **33**: 181–183

Fosså SD, Oliver RT, Stenning SP et al (1997) Prognostic factors for patients with advanced seminoma treated with platinum-based chemotherapy. *Eur J Cancer* **33**: 1380–1387

Horwich A and Dearnaley DP (1992) Treatment of seminoma. *Semin Oncol* **19**: 171–180

Horwich A, Dearnaley DP Duchesne GM et al (1989) Simple nontoxic treatment of advanced metastatic seminoma with carboplatin. *J Clin Oncol* **7**: 1150–1156

*© 2000 Cancer Research Campaign*

Horwich A, Sleijfer DT Fossa SD et al (1997) Randomized trial of bleomycin, etoposide, and cisplatin compared with bleomycin, etoposide, and carboplatin in good-prognosis metastatic nonseminomatous germ cell cancer: a Multiinstitutional Medical Research Council/European Organization for Research and Treatment of Cancer Trial. *J Clin Oncol* **15**: 1844–1852

Loehrer PJ, Johnson D, Elson P et al (1995) Importance of bleomycin in favourable-prognosis disseminated germ cell tumors: An Eastern Cooperative Oncology Group Trial. *J Clin Oncol* **13**: 470–476

Logothetis CJ, Samuels ML, Ogden SL et al (1987) Cyclophosphamide and sequential Cisplatin for advanced seminoma: long-term follow-up in 52 patients. *J Urol* **138**: 789–794

Mencel PJ, Motzer RJ, Mazumdar M et al (1994) Advanced seminoma: treatment results, survival, and prognostic factors in 142 patients. *J Clin Oncol* **12**: 120–126

Motzer RJ, Bosl GJ, Geller NL et al (1988) Advanced seminoma: the role of chemotherapy and adjunctive surgery. *Ann Intern Med* **108**: 513–518

Oliver RTD (1988) Long-term follow-up of single agent cisplatin in metastatic seminoma and surveillance for Stage I seminoma. Presented at the 24th Annual Meeting, ASCO. *Proc Am So Clin Oncol* **7**: 120

Oliver RTD, Edmonds PM, Ong JYH et al (1994) Pilot studies of 2 and 1 course carboplatin as adjuvant for stage I seminoma: should it be tested in a randomized trial against radiotherapy? *Int J Radiat Oncol Biol Phys* **29**: 3–8

Peckham MJ, McElwain TJ, Barrett A et al (1979) Combined management of the malignant teratoma of the testis. *The Lancet* **1**: 267–270

Puc HS, Heelan R, Mazumdar M et al (1996) Management of residual mass in advanced seminoma: Results and recommendations from the Memorial Sloan-Kettering Cancer Center. *J Clin Oncol* **14**: 454–460

Samuels ML, Lanzotti VJ, Holoye PY et al (1976) Combination chemotherapy in germinal cell tumor. *Cancer Treat Rev* **3**: 185–204

Samuels ML and Logothetis CJ (1983) Follow-up study of sequential weekly pulse Cis-platinum for advanced seminoma. *Proc Am Soc Clin Oncol* **2**: 137

Schmoll H-J, Bokemeyer C, Harstrick A et al (1991) Single agent carboplatinum (CBDCA) for advanced seminoma: A phase II study. *Proc Am Soc Clin Oncol* **10**: 181

Schultz SM, Einhorn LH, Conces DJJ et al (1989) Management of postchemotherapy residual mass in patients with advanced seminoma: Indiana University Experience. *J Clin Oncol* **7**: 1497s–1503

### APPENDIX

Other study participants:

| | |
|---|---|
| J Bozzino and H Lucraft | Northern Centre for Cancer Treatment, Newcastle |
| P I Clark | Clatterbridge Hospital, Liverpool |
| M Cornbleet and G W Howard | Western General, Edinburgh |
| T Ganesan | Churchill Hospital, Oxford |
| R J Grieve and D Jones | Walsgrave Hospital, Coventry |
| P Harper | Guy’s Hospital, London |
| N Hodson | Royal Sussex County Hospital, Brighton |
| S B Kaye and H Yosef | Glasgow Royal Infirmary |
| R Phillips | Westminster Hospital |
| W Pratt | Essex County Hospital |
| G Read and R D James | Christie Hospital, Manchester |
| G J S Rustin | Mt Vernon Hospital, Middlesex |
| D Whillis | Raigmore Hospital, Inverness |
| P H M de Mulder | University Hospital, Nijmegen |
| R de Wit | Daniel den Hoed Kliniek, Rotterdam |
| H J Keizer and J Maartense | Academic Ziekenhuis, Leiden |
| J Croles | Willem Alexander Ziekenhuis’s Hertogenbosch |

*© 2000 Cancer Research Campaign*
